# Plasma cell‐free DNA quantification is highly correlated to tumor burden in children with neuroblastoma

**DOI:** 10.1002/cam4.1586

**Published:** 2018-06-14

**Authors:** Xisi Wang, Lijun Wang, Yan Su, Zhixia Yue, Tianyu Xing, Wen Zhao, Qian Zhao, Chao Duan, Cheng Huang, Dawei Zhang, Mei Jin, Xianfeng Cheng, Shenglan Chen, Yi Liu, Xiaoli Ma

**Affiliations:** ^1^ Beijing Key Laboratory of Pediatric Hematology Oncology National Discipline of Pediatrics Ministry of Education, MOE Key Laboratory of Major Diseases in Children Hematology Oncology Center Beijing Children's Hospital National Center for Children's Health Capital Medical University Beijing China; ^2^ Beijing Keyin Technology Company Beijing Keyin Evergreen Institutes for Medical Research Company Limited Chaoyang District, Beijing China; ^3^ Taizhou Genewill Medical Laboratory Company Limited Pharmaceutics City of China Taizhou Jiangsu China

**Keywords:** biomarker, minimal residual disease, neuroblastoma, plasma cell‐free DNA, tumor burden

## Abstract

To evaluate plasma cell‐free DNA (cfDNA) as a promising biomarker for neuroblastoma (NB) tumor burden. Seventy‐nine eligible patients with newly diagnosed NB were recruited from Beijing Children's Hospital between April 2016 and April 2017. Additionally, from September 2011 to June 2017, 79 patients with stable NB were evaluated with a median follow‐up time of 21 months. Approximately 2 mL of peripheral blood was drawn upon enrollment, and plasma cfDNA levels were measured via quantitative polymerase chain reaction (qPCR). Total cfDNA analysis was performed using the long interspersed nuclear element 1 (LINE‐1) 79 bp fragment, and DNA integrity was calculated by the ratio of the LINE‐1 300 bp fragment to the LINE‐1 79 bp fragment. A total of 79 NB patients with a median age of 36 months comprised the group of newly diagnosed NB patients. The main primary tumor site was the retroperitoneal and adrenal region (81%). Three or more metastatic sites were found in 17.7% of patients. Stable NB patients older than 18 months comprised 98.7% of the stable NB patients. Neuron‐specific enolase (NSE), lactate dehydrogenase (LDH), and cfDNA levels were dramatically increased in the newly diagnosed NB patients and significantly different from those in the stable NB patients. Moreover, the concentration of cfDNA was much higher in patients with larger tumors. By analyzing the area under the receiver operator characteristic (ROC) curve (AUC), the areas of total cfDNA, NSE, and LDH levels were 0.953, 0.929, and 0.906, respectively. The sensitivity and specificity data clarified that the level of circulating cfDNA in plasma can be considered as a reliable biomarker for describing tumor load in NB. The plasma cfDNA concentration was as good as the levels of LDH and NSE to discriminate the tumor burden in children with NB.

## 1 INTRODUCTION

Neuroblastoma (NB) is an embryonal tumor that arises from the sympathetic nervous system and represents the most frequently diagnosed malignancy in the first year of life. Although NB patients who are stratified into the low and intermediate risk groups exhibit an excellent prognosis, the long‐term survival rate of high‐risk patients remains <50%.^1^, ^2^ This survival rate is mainly due to tumor relapse or regrowth caused by the activation of chemoresistant minimal residual disease (MRD).^1^, ^3^, ^4^, ^5^ To evaluate the therapeutic response and disease status of NB patients, several MRD detection methods, including ^131^I‐metaiodobenzylguanidine (^131^I‐MIBG), have a high detection sensitivity in NB primary tumors and bone or bone marrow metastasis.^6^, ^7^ However, ^131^I‐MIBG detection is complicated and requires a long time. Therefore, the clinical evaluation of a precise diagnosis of MRD by molecular pathology in NB patients remains to be established.

Plasma cell‐free DNA (cfDNA) detection has been widely studied in malignant tumors but not in pediatric tumors,^8^ such as NB. It is important and necessary to determine whether plasma cfDNA could serve as a biomarker for NB. Based on others reports,^9^, ^10^, ^11^, ^12^, ^13^, ^14^ it is feasible to introduce plasma cfDNA to evaluate the tumor burden in NB patients. The method is reproducible and convenient. The present work facilitates the diagnosis and improves measurement of NB tumor burden. We selected plasma cfDNA as a biological marker to detect the MRD in NB patients and evaluated the clinical value by quantitative polymerase chain reaction (qPCR) combined with clinical features and imaging results. Our aim is to establish a molecular biological method for MRD detection and provide a basis for detecting and improving NB treatments.

## 2 MATERIAL AND METHODS

### 2.1 Patients

Seventy‐nine eligible patients with newly diagnosed NB were recruited in the Hematology Oncology Center, at Beijing Children's Hospital, Capital Medical University, between April 2016 and April 2017. From September 2011 to June 2017, 79 patients with stable NB were monitored and evaluated with a median follow‐up time of 21 months (ranged from 9 to 64 months). The stable disease status of NB was described by multiple examinations. First, 2 independent microscopic examinations confirmed no NB cells in bone marrow. Second, independent pathological experts confirmed no progression via radiography. Third, 2 serum tumor markers, lactate dehydrogenase (LDH) and neuron‐specific enolase (NSE), were down‐regulated in the stable disease but upregulated in all newly diagnosed NB.

This research and the BCH‐NB‐2007 protocol were approved by the Beijing Children's Hospital Institutional Ethics Committee (No. 2016‐65). Informed consent was obtained from the parents or guardians of each patient according to the Declaration of Helsinki.

### 2.2 Diagnostics and staging systems

Microscopic examinations of bone marrow aspirates and biopsies were performed to determine the presence of NB cells. Serum tumor markers such as LDH and NSE levels were detected at the time of diagnosis in all patients and at the time of stable disease evaluation for the diagnosis and monitoring of NB. The defining characteristics of high‐risk NB (HR‐NB) include an age of more than 18 months, metastases (International Neuroblastoma Staging System 4, INSS‐4) or *MYCN* gene amplification. The BCH‐NB‐2007‐HR protocol was based on the Hong Kong Pediatric Hematology and Oncology Study Group (HKPHOSG)^15^ and a German report.^1^

### 2.3 Sample processing

Venous blood samples were collected in EDTA‐coated tubes and plasma was separated by centrifugation at 1600 *g* for 10 minutes. Supernatant was transferred to a new tube and centrifuged at 16 000 *g* for 10 minutes. Purified plasma was carefully removed without disturbing the lower residual layer and used for DNA extraction immediately or stored at −80°C.

### 2.4 DNA purification

Plasma samples were thawed on ice and centrifuged at 10 000 *g* for 3 minutes before DNA purification. DNA was purified from 200 μL of plasma and eluted by 300 μL of elution buffer using QIAamp DNA Blood Mini Kits (Qiagen, Valencia, CA, USA) according to the manufacturer's instructions. DNA samples were ready to use for quantification or stored at −20°C.

### 2.5 Quantitative polymerase chain reaction

Quantitative polymerase chain reaction was performed on a LightCycler LC480 PCR machine (Roche Molecular Systems, Inc. Pleasanton, CA, USA). To measure the concentration of plasma cfDNA, the repetitive LINE‐1 (long interspersed nuclear element 1) 79 bp and LINE‐1 300 bp DNA fragments were amplified as described, respectively.^16^, ^17^ The LINE‐1 79 bp primers amplified apoptotic and nonapoptotic DNA fragments while the LINE‐1 300 bp primers amplified nonapoptotic DNA fragments only.

The total amount of plasma DNA was represented by the qPCR result with LINE‐1 79 bp primers. The DNA integrity index was calculated as the ratio of the LINE‐1 300 and LINE‐1 79 qPCR results. A serially diluted standardized solution of human genomic DNA (Thermo Fisher Scientific, Waltham, MA, USA) was used to create a reference standard curve. The concentration of cfDNA in each sample was calculated according to the standard curve. The qPCR reactions were performed in triplicate, and mean values of the triplicates were used for further analysis. The qPCR reaction mixture was 10 μL and contained 2 μL of the eluted DNA, 1 μL (final concentration 0.2 μm) of each forward and reverse primer of LINE‐1 79 or LINE‐1 300, 5 μL of UltraSYBR Mixture (Cwbiotech, Beijing, China) and 1 μL of double‐distilled water. Cycling conditions were 1 minutes at 95°C and 35 cycles of 95°C for 8 seconds and 60°C for 15 seconds. Each plate contained a plasma DNA sample, a negative control (water template) and 7 serially diluted standard DNA solutions (10, 5, 1, 0.5, 0.25, 0.0625, 0.015 ng/μL).

### 2.6 Statistics

Statistical analysis was performed in R statistical environment (R‐version 3.4.0) and included Mann‐Whitney *U* tests, one‐way ANOVAs, boxplots and ROC analysis (Bioconductor ROC package). A *P*‐value lower than .05 was considered statistically significant.

## 3 RESULTS

### 3.1 Clinical characters and demographic information

A total of 79 pediatric patients aged 1‐148 months (median, 36 months) with newly diagnosed NB were enrolled. As shown in Table 1, children aged from 18 to 60 months accounted for 60.8% of the patients. There were 1.47‐fold more male than female patients. Eighty‐one percent of primary tumor sites were detected in the retroperitoneal and adrenal region while other locations accounted for only 19%, including the thorax, neck, and others. In addition, most patients were categorized into stage IV in the clinic. Approximately 50.6% of the NB patients had 1 or 2 metastatic sites, while 31.6% had 3 sites. NB with metastasis in more than 3 organs comprised 17.7% of the patients. The most frequent metastatic sites were bone, bone marrow, and distant lymph nodes, which occupied more than 60% of the metastatic patients. *MYCN* copies were amplified in 19% of patients. According to the clinical serum test, more NB patients showed below 370 ng/mL NSE (58.2%) or lower than 1500 IU/L of LDH (78.5%). However, the numbers of patients with LDH lower than 500 IU/L and from 500~1500 IU/L were similar.

**Table 1:** Demographic and clinical features of patients with newly diagnosed NB (N = 79)

Characteristics	Total cases, N (%)
Age (mo)	
<18	19 (24.1)
≥18 and <60	48 (60.8)
≥60	12 (15.2)
Sex	
Female	32 (40.5)
Male	47 (59.5)
Primary site	
Abdomen	64 (81.0)
Thorax and other	15 (19.0)
Tumor size	
>10 cm	42 (53.2)
≤10 cm	37 (46.8)
Tumor stage	
II/III	15 (19.0)
IV	64 (81.0)
*MYCN* gene	
Amplification	15 (19.0)
Nonamplification	64 (81.0)
NSE (ng/mL)	
<370	46 (58.2)
≥370	33 (41.8)
LDH (IU/L)	
≤500	32 (40.5)
>500 and <1500	30 (38.0)
≥1500	17 (21.5)
Metastatic site	
Bone	51 (64.6)
Bone marrow	52 (65.8)
Distant lymph node	49 (62.0)
Liver	14 (17.7)
Central nervous system	12 (15.2)
Number of organs with metastasis	
<3	40 (50.6)
=3	25 (31.6)
>3	14 (17.7)

LDH, lactate dehydrogenase; NB, neuroblastoma; NSE, neuron‐specific enolase.

Intriguingly, the number of patients was similar based on the tumor size classification. As measured among 79 newly diagnosed NB children, the minimum and maximum diameters of tumors were 1.5 and 21.8 cm, respectively. According to the diameter median of 10.5 ± 4.78 cm, newly NB patients were divided into 2 groups (>10 and ≤10 cm).There were 42 NB kids whose tumor diameter more than 10 cm while 37 NB kids not. The median of tumor size was 6.7 ± 2.7 cm and 13.2 ± 2.97 in NB patients with smaller or larger tumors, respectively (Table S1).

Seventy‐nine NB patients with stable disease were monitored from 9 to 64 months and validated according to clinical examination by the end time point of surveillance. In Table 2, these data show that the demographic information was similar in newly diagnosed and stable NB patients, including sex and disease origin. There were more boys than girls and abdominal tumors were predominantly found in the stable NB patients. According the treatment regimen, these NB patients were divided into follow‐up and retinoic acid groups with approximately 60.8% and 39.2% of the patients, respectively. The stable NB patients older than 18 months comprised 98.7% of the stable NB patients, and these children were distributed equally with respect to the 60‐months threshold.

**Table 2:** Demographic and clinical features of patients with stable NB ranging from 9 to 64 mo (N = 79)

Characteristics	Total cases, N (%)
Age (mo)	
<18	1 (1.3)
≥18 and <60	38 (48.1)
≥60	40 (50.6)
Sex	
Female	37 (46.8)
Male	42 (53.2)
Primary site	
Abdomen	55 (69.6)
Thorax and other	24 (30.4)
Treatment regimen	
Follow‐up	48 (60.8)
Retinoic acid	31 (39.2)

### 3.2 Performance comparisons among total plasma cfDNA concentration, NSE and LDH as tumor burden biomarkers of NB

Our investigation recorded stable disease NB clinical data from the blood analysis, including LDH, NSE, and cfDNA concentrations (Table 3). First, cfDNA was measured in stable disease patients to detect the MRD. Next, the median concentrations of NSE, LDH, and cfDNA were very similar regardless of the status described, such as age, sex, primary original sites, or therapy regime during monitoring time window.

**Table 3:** Serum NSE and LDH levels and total plasma cfDNA in the stable disease NB group

Characteristics	N	NSE median (min‐max ng/mL)	LDH median (min‐max IU/L)	cfDNA median (min‐max ng/mL)	cfDNA integrity median (min‐max)
Age (mo)					
<18	1	28.7 (28.7‐28.7)	261 (261‐261)	8.85 (8.85‐8.85)	0.24 (0.24‐0.24)
≥18 and <60	38	25.20 (15.0‐84.3)	275 (68‐454)	7.03 (3.02‐28.17)	0.22 (0.10‐0.41)
≥60	40	22.85 (16.2‐52.4)	256 (167‐379)	6.445 (2.44‐24.53)	0.21 (0.12‐0.35)
Sex					
Female	37	23.5 (15.0‐52.7)	271 (195‐454)	6.74 (2.96‐25.07)	0.21 (0.10‐0.41)
Male	42	23.95 (16.5‐84.3)	260.5 (68‐379)	6.855 (2.44‐28.17)	0.23 (0.12‐0.39)
Primary site					
Abdomen	55	23.7 (16.2‐84.3)	268 (195‐454)	6.80 (2.44‐28.17)	0.21 (0.10‐0.35)
Thorax and other	24	23.7 (15.0‐52.4)	265 (68‐365)	7.12 (2.96‐24.53)	0.24 (0.12‐0.41)
Maintenance therapy					
None	48	23.6 (15.0‐52.4)	265.5 (68‐454)	6.635 (2.44‐24.53)	0.23 (0.10‐0.41)
Retinoic acid	31	23.9 (16.5‐84.3)	268 (195‐369)	6.91 (3.34‐28.17)	0.21 (0.13‐0.35)

**Figure 1:**
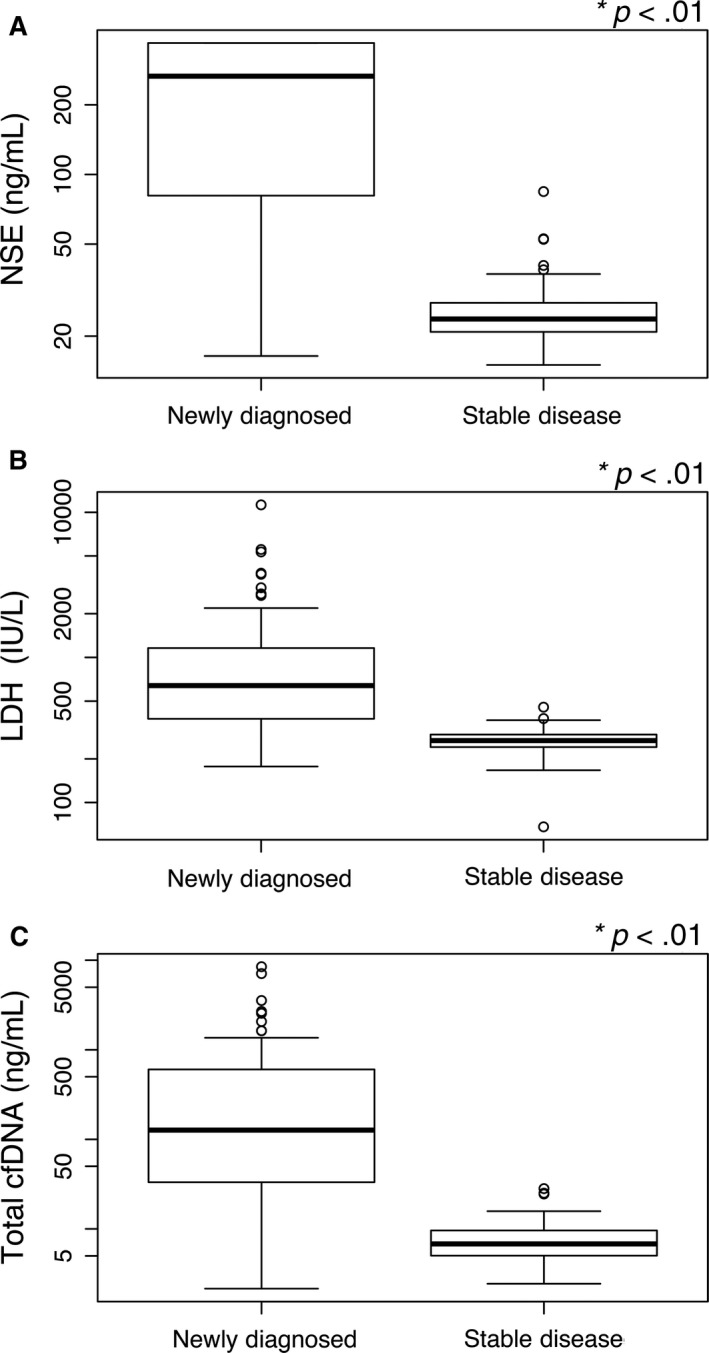
Comparison of NSE, LDH, and cfDNA level between newly diagnosed NB patients and stable NB. Box plots for comparison of biomarker levels between groups. Levels of NSE (A), LDH (B), and total plasma cfDNA (C) in the newly diagnosed NB patients were significantly higher in comparison with the stable NB (*P* < .01). (*Mann‐Whitney *U* test)

Usually, without any treatment, newly diagnosed NB patients have a more severe tumor load than those with stable NB.^18^, ^19^, ^20^ Therefore, the stable disease NB patients were considered as the tumor burden control in comparison to the newly diagnosed patients in the clinical evaluations. In Figure 1, as expected, NSE levels were approximately 11.2‐fold higher in the newly diagnosed patients than in the stable disease patients (265.80 ± 139.08 vs 23.70 ± 23.90 ng/mL; *P *<* *.01; Figure 1A). The trend of the LDH levels was similar to that of the NSE levels (639.00 ± 1563.09 vs 267.50 ± 74.90 IU/L; *P *<* *.01; Figure 1B). Consistently, the median total plasma DNA concentration was 18.6 times higher in the newly diagnosed patients than in the stable disease NB patients (126.71 ± 1344.67 ng/mL vs 6.83 ± 5.99 ng/mL; *P *<* *.01; Figure 1C). All the 3 datasets indicated that the NSE, LDH, and cfDNA concentrations were dramatically increased in newly diagnosed NB patients while lower levels were maintained in the stable disease NB patients.

There were some newly diagnosed NB children whose NSE or LDH were in lower level but cfDNA was kept high. Subjectively, newly diagnosed NB patients with NSE less than 50 ng/mL were 16. However, only in 1 NB patient, cfDNA level was dramatically lower, 2.15 ng/mL. Other 15 patients’ cfDNA levels were higher than 30 ng/mL, which was the top level in stable NB. Moreover, the median of cfDNA was 155.32 ng/mL, that was higher than the median of all newly diagnosed NB patients (126.71 ng/mL). In parallel, 32 newly diagnosed NB were found with LDH less than 500 IU/L (range from 177 to 494 IU/L). There were 7 kids whose cfDNA level <30 ng/mL while 25 kids not. The median of cfDNA from 25 NB was 186.62 ng/mL, higher than it from total newly NB too. The analyzed data indicated that some newly diagnosed NB had lower levels of NSE and/or LDH while their cfDNA level was high in accordance with tumor load (Table S2).

### 3.3 Correlation between plasma cfDNA levels and clinical characteristics

For the newly diagnosed NB group, associations between the level of total plasma cfDNA and other established clinical parameters were analyzed and are presented in Table 4. Except for the age distribution, the significant differences were all significant in the other parameter categories (*P *<* *.05), and the mean cfDNA concentration was obviously upregulated in the NB patients with heavier tumor loads. For instance, the most significant difference in cfDNA was 32.4 times more (2020.4 vs 62.3 ng/mL) in NB patients with high‐level LDH (≥1500 IU/L) than those with low‐level LDH (≤500 IU/L). When comparing patients by other parameters, the cfDNA levels were also 9‐13 times greater in the high‐risk NB patients compared to the low‐risk NB patients, including patients with >3 metastatic organ locations compared to those with <3 metastatic organs (1647.3 vs 131.3 ng/mL), patients with *MYCN* amplification compared to those with nonamplification (1387 vs 411.6 ng/mL) and patients with NSE level = 370 ng/mL compared to those with NSE < 370 ng/mL (1165 vs 189.2 ng/mL). Concerning tumor sizes and clinical stages, the cfDNA ratios were approximately 6 between the patients with abdomen tumors and those with other primary tumor sites (708.7 vs 119.6 ng/mL) and between stage IV patients and patients with earlier stages (1465.5 vs 113.6 ng/mL). The smallest fold changes were approximately 3 between male and female patients (829.4 vs 255.3 ng/mL) and between patients with tumor sizes >10 cm and those with tumor sizes ≤10 cm (861.8 vs 296 ng/mL). Thus, the cfDNA concentration in the newly diagnosed NB patients was significantly higher in the high‐risk group (Table 4).

**Table 4:** Total plasma cfDNA levels in newly diagnosed NB patients

Characteristics	N	Mean ± SD	*P*‐values
Age (mo)			
<18	19	104.4 ± 176.2	.0566^a^
≥18 and <60	48	885.0 ± 1658.6	
≥60	12	223.7 ± 288.9	
Sex			
Female	32	255.3 ± 414.3	.03868^b^
Male	47	829.4 ± 1677.5	
Primary site			
Abdomen	64	708.7 ± 1469.7	.001345^b^
Thorax and other	15	119.6 ± 229.0	
Tumor size			
>10 cm	42	861.8 ± 1738.15	.01328^b^
≤10 cm	37	296.0 ± 549.5	
Tumor stage			
II/III	15	113.6 ± 317.5	.00004^b^
IV	64	710.1 ± 1465.5	
*MYCN* gene			
Amplification	15	1387.0 ± 1957.1	.01356^b^
Nonamplification	64	411.6 ± 1097.3	
NSE (ng/mL)			
<370	46	189.2 ± 429.5	3.141e‐08^b^
≥370	33	1165.0 ± 1891.1	
LDH (IU/L)			
≤500	32	62.3 ± 90.2	4.08e‐07^a^
>500 and <1500	30	360.4 ± 328.4	
≥1500	17	2020.4 ± 2402.9	
Number of organs with metastasis			
<3	40	131.3 ± 287.2	.000964^a^
=3	25	780.9 ± 1598.3	
>3	14	1647.3 ± 1944.2	

a One‐way ANOVA.

b Mann‐Whitney *U* Test.

### 3.4 Receiver operator characteristic (ROC) curve analysis of cfDNA levels in NB patients

**Figure 2:**
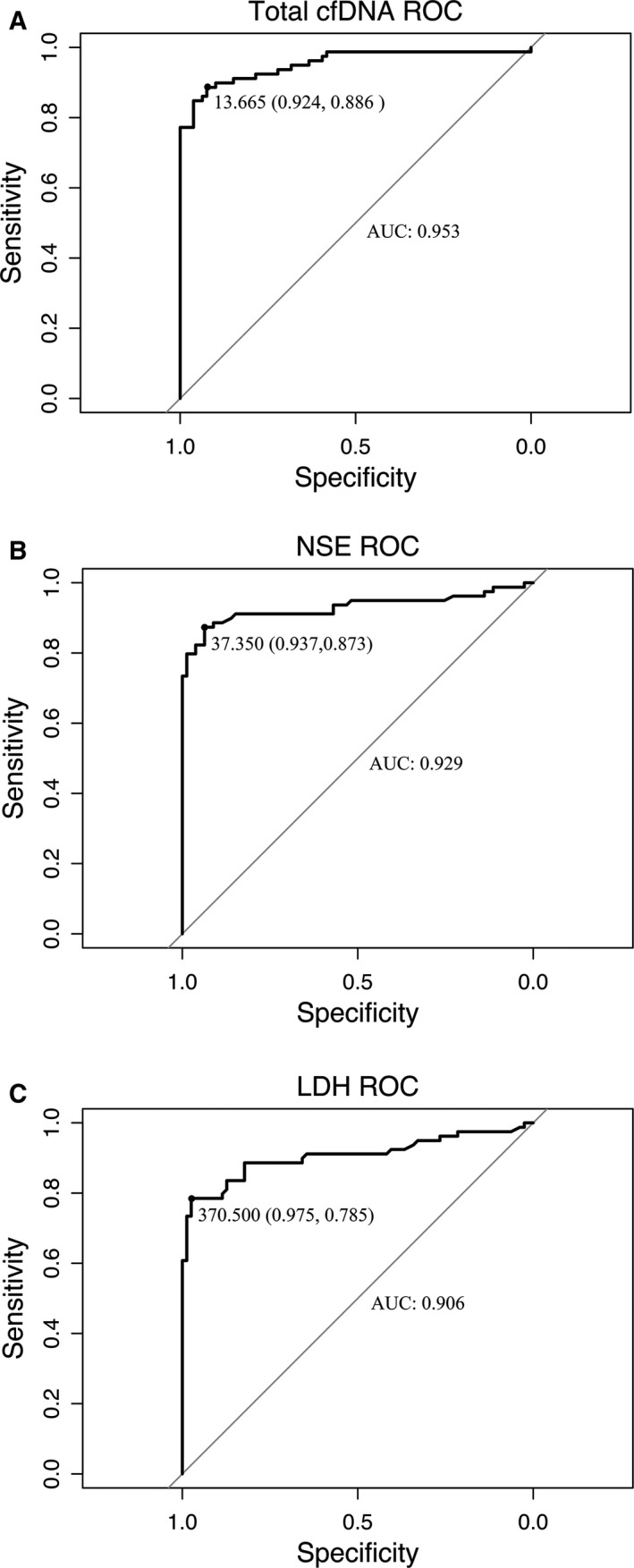
Receiver operating characteristic (ROC) curves for distinguishing between newly diagnosed NB patients from stable NB. A, The area under the receiver operator characteristic (ROC) curve (AUC) of total plasma cfDNA was 0.953, with 88.6% sensitivity and 92.4% specificity at 13.665 ng/mL. B, AUC of NSE was 0.929, with 87.3% sensitivity and 93.7% specificity at 37.350 ng/mL. C, AUC of LDH was 0.906, with 78.5% sensitivity and 97.5% specificity at 370.50 IU/L

By analyzing the area under the ROC curve (AUC) between the newly diagnosed and stable disease NB patients (Figure 2), the total plasma cfDNA levels showed that the AUC value was 0.953 and represented a significant, positive correlation with the confirmation of newly diagnosed NB. Meanwhile, the assessed AUC values of NSE and LDH were 0.929 and 0.906, respectively. All 3 AUC values were higher than 0.9, suggesting a comparable and discriminating ability to distinguish newly diagnosed NB from stable disease NB patients. The AUC of the cfDNA concentration was the best molecular assay to measure NB tumor burden followed by NSE and LDH. However, the AUC value of combined cfDNA and NSE or LDH did not improve the discrimination ability (data not shown).

### 3.5 DNA integrity and cfDNA performance between newly diagnosed and stable NB patients

**Figure 3:**
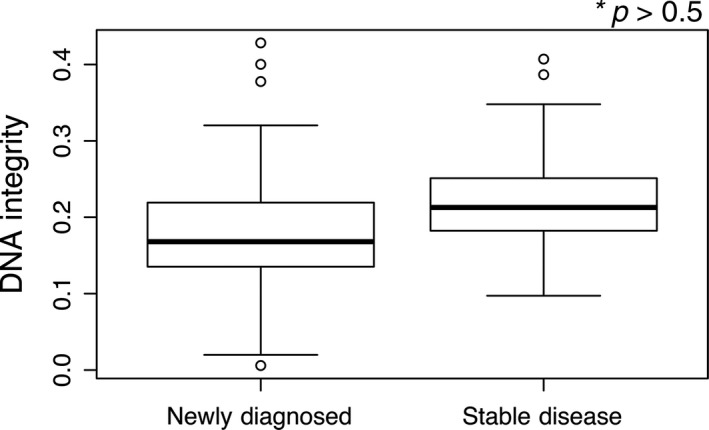
Comparison of DNA integrity between newly diagnosed NB patients and stable NB. Box plots for comparison of DNA integrity in the newly diagnosed NB patients were not significantly different in comparison with the stable NB (*P* > .05). (*Mann‐Whitney *U* test)

The values of DNA integrity established by the ratio LINE‐1 300/LINE‐1 79 ranged from 0.21 to 0.24 in stable disease NB patients (Table 3). Similar to the total cfDNA levels in stable NB patients, the ratios representing DNA integrity were very close to each other among different subpopulation categories. In parallel, the difference in DNA integrity between the newly diagnosed and stable NB patients was 0.17 vs 0.21, and there was no statistical significance (Figure 3, *P* > .05). Furthermore, the DNA integrity was not significantly different based on the clinical variables in newly diagnosed NB patients, including age, sex, clinical stages, *MYCN* amplification, metastasis, etc. The differences in DNA integrity between primary tumor sites and between tumor volumes were significant in newly diagnosed NB patients (Table 5). For example, the DNA integrity from abdominal tumors was significantly less than that from other primary sites (0.17 vs 0.21). More intriguingly, tumors with a larger diameter displayed a lower DNA integrity ratio than those with a smaller volume (0.16 vs 0.20). Consistently, higher levels of NSE and LDH were accompanied by smaller DNA integrities (0.15 vs 0.20; 0.12 vs 0.19; respectively). DNA was more fragmented in the NB patients with a heavier tumor burden, as determined by tumor origins, tumor volume, and tumor serum biomarkers.

**Table 5:** DNA integrity index according to clinical and genetic characteristics in newly diagnosed NB patients

Characteristics	N	Mean ± SD	*P*‐values
Age (mo)			
<18	19	0.19 ± 0.10	.529^a^
≥18 and <60	48	0.17 ± 0.07	
≥60	12	0.20 ± 0.04	
Sex			
Female	32	0.18 ± 0.06	.97^b^
Male	47	0.18 ± 0.08	
Primary site			
Abdomen	64	0.17 ± 0.08	.02253^b^
Thorax and other	15	0.21 ± 0.05	
Tumor size			
>10 cm	42	0.16 ± 0.08	.02549^b^
≤10 cm	37	0.20 ± 0.07	
Tumor stage			
II/III	15	0.20 ± 0.09	.175^b^
IV	64	0.17 ± 0.07	
*MYCN* gene			
Amplification	15	0.16 ± 0.07	.4495^b^
Nonamplification	64	0.18 ± 0.08	
NSE (ng/mL)			
<370	46	0.20 ± 0.08	.01219^b^
≥370	33	0.15 ± 0.06	
LDH (IU/L)			
≤500	32	0.19 ± 0.08	.00316^a^
>500 and <1500	30	0.19 ± 0.06	
≥1500	17	0.12 ± 0.06	
Number of organs with metastasis			
<3	40	0.19 ± 0.08	.197^a^
=3	25	0.17 ± 0.06	
>3	14	0.16 ± 0.08	

a One‐way ANOVA.

b Mann‐Whitney *U* Test.

## 4 DISCUSSION

Recently, emerging evidence has elucidated that the absolute cfDNA concentration and DNA integrity might be candidate biomarkers for the diagnosis and prognosis of malignant tumors.^8^, ^10^, ^13^, ^14^, ^21^, ^22^ Compared to patients with benign tumors and healthy controls, the concentrations of circulating cfDNA were significantly distinguished and higher in adult patients with various cancers, including lung cancer, colorectal cancer, and breast cancer. Moreover, a great number of researchers have demonstrated that plasma DNA could be applied to estimate the therapy response and prognosis of broad cancers in the clinic.^9^, ^10^, ^11^, ^12^, ^13^, ^14^ Generally, increased levels of cfDNA were closely correlated with exacerbation or poor outcomes in cancer patients. These studies indicated that circulating cfDNA could be applied to detect MRD as well as represent a surrogate biomarker of tumor burden in a variety of malignant tumors. However, reports related to circulating cfDNA are not widely documented in pediatric solid tumors, and sample sizes need to be expanded further.^23^ For instance, although there were not significant relationships between plasma cfDNA levels and tumor sizes or origins in 44 pediatric solid tumors, such as NB, hepatoblastoma, nephroblastoma, and rhabdomyosarcoma, cfDNA concentrations were higher in stage IV than in stages I‐III tumors, which represents a significant association between cfDNA quantification and clinical stages.^23^

To quantify cfDNA concentration and calculate DNA integrity from peripheral blood, qPCR is a popular method.^17^, ^24^, ^25^, ^26^, ^27^, ^28^, ^29^, ^30^, ^31^ In this study, LINE‐1 79 and 300 bp DNA fragments were selected to examine the total cfDNA concentration and DNA integrity in plasma.^16^, ^17^ In several studies, *MYCN* amplification has been used to predict or assay the effects of treatment and MRD of NB solid tumors in children.^18^, ^19^, ^20^, ^23^, ^32^ However, amplification of *MYCN* measured by PCR was detected in a relatively small proportion of blood samples from children with NB.^20^, ^23^ Therefore, cfDNA quantification could be an effective and reproducible approach to detect MRD in NB and other solid pediatric malignant tumors.

In malignant solid tumors, the DNA integrity index was also associated with tumor loads and is expected to be used as a molecular diagnostic biomarker in the clinic.^33^, ^34^, ^35^, ^36^ Debatably, the DNA integrity index was higher in breast cancer patients than in patients with benign tumors and healthy controls,^35^ while a higher DNA integrity is predicted to have a lower risk of recurrence.^33^ However, in colorectal cancer and hepatocellular cancer patients, DNA integrity was much lower than that in benign patients and healthy volunteers.^34^, ^36^ Thus, fragmentation of cfDNA represented a clue of genetic aberration for malignant solid tumors.

Our work found that the absolute concentration of cfDNA was closely, positively associated with NB tumor burden. In comparison to stable NB, cfDNA was remarkably upregulated in newly diagnosed NB (Figure 1). Larger tumor size and higher levels of cfDNA were detected in newly diagnosed NB patients (Table 4). In addition, the primary locations of tumors, clinical stages, and metastatic features were accompanied by an increased level of cfDNA in newly diagnosed NB patients (Table 4). Notably, higher levels of NSE and LDH were significantly correlated with the cfDNA concentration (Figure 2). Patients with a heavier NB tumor burden had higher levels of NSE, LDH, and cfDNA. The AUC calculation elucidated the power of cfDNA levels to discriminate the tumor burden of newly diagnosed NB from stable disease NB, and the AUC for cfDNA levels was better able to discriminate the tumor burden than the AUC of NSE or LDH levels (Figure 2). Unlike the total level of cfDNA, the DNA integrity index could not distinguish between newly diagnosed and stable NB (Figure 3). However, the measurable level of cfDNA in stable NB patients demonstrated that the MRD of NB could be examined (Table 3). Our data showed that cfDNA level could be considered as surrogate biomarker for NB tumor burden. However, quantity of cfDNA should be applied in clinic independently. In the future, whether cfDNA could be instead of the 2 serum biomarkers, further investigations need to be done. Next, we will investigate whether the quantification of plasma cfDNA, NSE, and LDH could be used to monitor therapy response and follow‐up. In progress, we are collecting data from these NB patients. Our work on dynamic therapy responses and following up in NB children will be presented in the future.

In conclusion, the quantification of plasma cfDNA was considered to be an effective and more precise biomarker to evaluate tumor burden as well as MRD of NB in a clinical setting.

## CONFLICT OF INTEREST

The authors declare that there are no conflicts of interest.

## References

[1] Berthold F , Spix C , Kaatsch P , Lampert F . Incidence, survival, and treatment of localized and metastatic neuroblastoma in Germany 1979‐2015. Paediatr Drugs. 2017;19:577‐593. 28786082 10.1007/s40272-017-0251-3 PMC5680382

[2] Irwin MS , Park JR . Neuroblastoma: paradigm for precision medicine. Pediatr Clin North Am. 2015;62:225‐256. 25435121 10.1016/j.pcl.2014.09.015

[3] Cheung NK , Ostrovnaya I , Kuk D , Cheung IY . Bone marrow minimal residual disease was an early response marker and a consistent independent predictor of survival after anti‐GD2 immunotherapy. J Clin Oncol. 2015;33:755‐763. 25559819 10.1200/JCO.2014.57.6777 PMC4334779

[4] van Wezel EM , Stutterheim J , Vree F , et al. Minimal residual disease detection in autologous stem cell grafts from patients with high risk neuroblastoma. Pediatr Blood Cancer. 2015;62:1368‐1373. 25939774 10.1002/pbc.25507

[5] Yamamoto N , Kozaki A , Hartomo TB , et al. Differential expression of minimal residual disease markers in peripheral blood and bone marrow samples from high‐risk neuroblastoma patients. Oncol Lett. 2015;10:3228‐3232. 26722317 10.3892/ol.2015.3710 PMC4665349

[6] DuBois SG , Mody R , Naranjo A , et al. MIBG avidity correlates with clinical features, tumor biology, and outcomes in neuroblastoma: a report from the Children's Oncology Group. Pediatr Blood Cancer. 2017;64:e26545 10.1002/pbc.26545 PMC5605392 28383813

[7] Polishchuk AL , Li R , Hill‐Kayser C , et al. Likelihood of bone recurrence in prior sites of metastasis in patients with high‐risk neuroblastoma. Int J Radiat Oncol Biol Phys. 2014;89:839‐845. 24867534 10.1016/j.ijrobp.2014.04.004

[8] Siravegna G , Marsoni S , Siena S , Bardelli A . Integrating liquid biopsies into the management of cancer. Nat Rev Clin Oncol. 2017;14:531‐548. 28252003 10.1038/nrclinonc.2017.14

[9] Ai B , Liu H , Huang Y , Peng P . Circulating cell‐free DNA as a prognostic and predictive biomarker in non‐small cell lung cancer. Oncotarget. 2016;7:44583‐44595. 27323821 10.18632/oncotarget.10069 PMC5190120

[10] Butler TM , Spellman PT , Gray J . Circulating‐tumor DNA as an early detection and diagnostic tool. Curr Opin Genet Dev. 2017;42:14‐21. 28126649 10.1016/j.gde.2016.12.003

[11] Coco S , Alama A , Vanni I , et al. Circulating cell‐free DNA and circulating tumor cells as prognostic and predictive biomarkers in advanced non‐small cell lung cancer patients treated with first‐line chemotherapy. Int J Mol Sci. 2017;18:pii: E1309. 10.3390/ijms18051035 PMC5454947 28492516

[12] Lan YT , Chen MH , Fang WL , et al. Clinical relevance of cell‐free DNA in gastrointestinal tract malignancy. Oncotarget. 2017;8:3009‐3017. 27936467 10.18632/oncotarget.13821 PMC5356859

[13] Li BT , Drilon A , Johnson ML , et al. A prospective study of total plasma cell‐free DNA as a predictive biomarker for response to systemic therapy in patients with advanced non‐small‐cell lung cancers. Ann Oncol. 2016;27:154‐159. 26487589 10.1093/annonc/mdv498 PMC4684155

[14] Tissot C , Toffart AC , Villar S , et al. Circulating free DNA concentration is an independent prognostic biomarker in lung cancer. Eur Respir J. 2015;46:1773‐1780. 26493785 10.1183/13993003.00676-2015

[15] Leung CK . Fifteen years’ review of advanced childhood neuroblastoma from a single institution in Hong Kong. Chin Med J (Engl). 1998;111:466‐469. 10374361

[16] Diehl F , Schmidt K , Choti MA , et al. Circulating mutant DNA to assess tumor dynamics. Nat Med. 2008;14:985‐990. 18670422 10.1038/nm.1789 PMC2820391

[17] Sunami E , Vu AT , Nguyen SL , Giuliano AE , Hoon DS . Quantification of LINE1 in circulating DNA as a molecular biomarker of breast cancer. Ann N Y Acad Sci. 2008;1137:171‐174. 18837943 10.1196/annals.1448.011

[18] Jung M , Russell AJ , Liu B , et al. A Myc activity signature predicts poor clinical outcomes in Myc‐associated cancers. Cancer Res. 2017;77:971‐981. 27923830 10.1158/0008-5472.CAN-15-2906

[19] Kang Z , Stevanovic S , Hinrichs CS , Cao L . Circulating cell‐free DNA for metastatic cervical cancer detection, genotyping, and monitoring. Clin Cancer Res. 2017;23:6856‐6862. 28899967 10.1158/1078-0432.CCR-17-1553 PMC7885032

[20] Yue ZX , Huang C , Gao C , et al. MYCN amplification predicts poor prognosis based on interphase fluorescence in situ hybridization analysis of bone marrow cells in bone marrow metastases of neuroblastoma. Cancer Cell Int. 2017;17:43. 28367105 10.1186/s12935-017-0412-z PMC5374581

[21] Hao TB , Shi W , Shen XJ , et al. Circulating cell‐free DNA in serum as a biomarker for diagnosis and prognostic prediction of colorectal cancer. Br J Cancer. 2014;111:1482‐1489. 25157833 10.1038/bjc.2014.470 PMC4200099

[22] Mittra I , Khare NK , Raghuram GV , et al. Circulating nucleic acids damage DNA of healthy cells by integrating into their genomes. J Biosci. 2015;40:91‐111. 25740145 10.1007/s12038-015-9508-6 PMC5779614

[23] Kurihara S , Ueda Y , Onitake Y , et al. Circulating free DNA as non‐invasive diagnostic biomarker for childhood solid tumors. J Pediatr Surg. 2015;50:2094‐2097. 26388126 10.1016/j.jpedsurg.2015.08.033

[24] Board RE , Williams VS , Knight L , et al. Isolation and extraction of circulating tumor DNA from patients with small cell lung cancer. Ann N Y Acad Sci. 2008;1137:98‐107. 18837931 10.1196/annals.1448.020

[25] Catarino R , Ferreira MM , Rodrigues H , et al. Quantification of free circulating tumor DNA as a diagnostic marker for breast cancer. DNA Cell Biol. 2008;27:415‐421. 18694299 10.1089/dna.2008.0744

[26] Jiang P , Chan CW , Chan KC , et al. Lengthening and shortening of plasma DNA in hepatocellular carcinoma patients. Proc Natl Acad Sci U S A. 2015;112:E1317‐E1325. 25646427 10.1073/pnas.1500076112 PMC4372002

[27] Kolesnikova EV , Tamkovich SN , Bryzgunova OE , et al. Circulating DNA in the blood of gastric cancer patients. Ann N Y Acad Sci. 2008;1137:226‐231. 18837952 10.1196/annals.1448.009

[28] Liao W , Mao Y , Ge P , et al. Value of quantitative and qualitative analyses of circulating cell‐free DNA as diagnostic tools for hepatocellular carcinoma: a meta‐analysis. Medicine (Baltimore). 2015;94:e722. 25860220 10.1097/MD.0000000000000722 PMC4554041

[29] van der Drift MA , Hol BE , Klaassen CH , et al. Circulating DNA is a non‐invasive prognostic factor for survival in non‐small cell lung cancer. Lung Cancer. 2010;68:283‐287. 19632736 10.1016/j.lungcan.2009.06.021

[30] Xue X , Teare MD , Holen I , Zhu YM , Woll PJ . Optimizing the yield and utility of circulating cell‐free DNA from plasma and serum. Clin Chim Acta. 2009;404:100‐104. 19281804 10.1016/j.cca.2009.02.018

[31] Yoon KA , Park S , Lee SH , Kim JH , Lee JS . Comparison of circulating plasma DNA levels between lung cancer patients and healthy controls. J Mol Diagn. 2009;11:182‐185. 19324991 10.2353/jmoldx.2009.080098 PMC2671334

[32] Yagyu S , Iehara T , Tanaka S , et al. Serum‐Based Quantification of MYCN Gene Amplification in Young Patients with Neuroblastoma: potential Utility as a Surrogate Biomarker for Neuroblastoma. PLoS ONE. 2016;11:e0161039. 27513929 10.1371/journal.pone.0161039 PMC4981470

[33] Cheng J , Cuk K , Heil J , et al. Cell‐free circulating DNA integrity is an independent predictor of impending breast cancer recurrence. Oncotarget. 2017;8:54537‐54547. 28903362 10.18632/oncotarget.17384 PMC5589601

[34] Huang A , Zhang X , Zhou SL , et al. Plasma Circulating Cell‐free DNA Integrity as a Promising Biomarker for Diagnosis and Surveillance in Patients with Hepatocellular Carcinoma. J Cancer. 2016;7:1798‐1803. 27698918 10.7150/jca.15618 PMC5039362

[35] Kamel AM , Teama S , Fawzy A , El Deftar M . Plasma DNA integrity index as a potential molecular diagnostic marker for breast cancer. Tumour Biol. 2016;37:7565‐7572. 26684805 10.1007/s13277-015-4624-3

[36] Yoruker EE , Ozgur E , Keskin M , Dalay N , Holdenrieder S , Gezer U . Assessment of circulating serum DNA integrity in colorectal cancer patients. Anticancer Res. 2015;35:2435‐2440. 25862911

