# Supplementary material for: Plasma cell‐free DNA quantification is highly correlated to tumor burden in children with neuroblastoma
Source: Cancer Med. 2018 Jun 14;7(7):3022–30. doi: 10.1002/cam4.1586 (PMC6051223; doi:10.1002/cam4.1586)
Supplement: Supplementary file 1 [file CAM4-7-3022-s001.docx]

Table S1

Tumor size distribution in newly diagnosed NB

| Tumor | N | Median ± SD (cm) | Minimum – Maximum (cm) |
| --- | --- | --- | --- |
| Total | 79 | 10.5 ± 4.78 | 1.5 – 21.8 |
| ≤10 cm | 37 | 6.7 ± 2.7 | 1.5 - 10 |
| >10 cm | 42 | 13.2 ± 2.97 | 10.2 – 21.8 |

Table S2

Lower NSE or LDH with increased cfDNA in newly diagnosed NB

| Quantity | N | Median ± SD | Minimum - Maximum |
| --- | --- | --- | --- |
| NSE < 50 ng/ml | 16 | 29.95 ± 9.07 | 16.4 – 44.5 |
| Total cfDNA (ng/ml) | 16 | 153.46 ± 1023.88 | 2.15 - 3550 |
| Total cfDNA < 30 ng/ml | 1 | 2.15 | 2.15 |
| Total cfDNA ≥ 30 ng/ml | 15 | 155.32 ± 1047.88 | 31.41 - 3550 |
| LDH <500 IU/L | 32 | 338 IU/L ± 78.18 | 177 IU/L - 494 |
| Total cfDNA (ng/ml) | 32 | 122.38 ± 864.52 | 2.15 – 3550 |
| Total cfDNA <30 ng/ml | 7 | 9.33 ± 6.08 | 2.15 – 21.21 |
| Total cfDNA ≥30 ng/m | 25 | 186.62 ± 940.93 | 30.22 - 3550 |
